# Assessment of shear wave elastography for evaluating neoadjuvant chemotherapy efficacy in breast cancer: correlation of stiffness changes with pathological response

**DOI:** 10.3389/fonc.2026.1839381

**Published:** 2026-10-26

**Authors:** Hongying Yang, Shan Liu, Xiaomao Luo, Zeng Zhang, Qin Su, Wenjing Pu, Haitao Chen, Qian An, Xin Liu, Likun Luan, Ningxin Zhang, Peijun Yao, Yingxian Zhang, Lei Zhang, Jie Yu, Zhiyao Li, Zhirui Chuan

**Affiliations:** 1 Department of Ultrasound, The Third Affiliated Hospital of Kunming Medical University, Yunnan Cancer Hospital, Peking University Cancer Hospital Yunnan, Kunming, Yunnan, China; 2 Department of Integrated Traditional Chinese and Western Medicine, The Third Affiliated Hospital of Kunming Medical University, Yunnan Cancer Hospital, Peking University Cancer Hospital Yunnan, Kunming, Yunnan, China; 3 Department of Otolaryngology, Kunming First People’s Hospital, Kunming, Yunnan, China; 4 Yunnan Institute of Traditional Chinese Medicine, Yunnan University of Chinese Medicine, Kunming, Yunnan, China; 5 Department of Pathology, The Third Affiliated Hospital of Kunming Medical University, Yunnan Cancer Hospital, Peking University Cancer Hospital Yunnan, Kunming, Yunnan, China; 6 Department of Gastric and Small Intestinal Surgery, The Third Affiliated Hospital of Kunming Medical University, Yunnan Cancer Hospital, Peking University Cancer Hospital Yunnan, Kunming, Yunnan, China; 7 Department of Thyroid and Breast Surgery Department, The Second Affiliated Hospital of Kunming Medical University, Kunming, Yunnan, China; 8 Department of Colorectal Surgery, The Third Affiliated Hospital of Kunming Medical University, Yunnan Cancer Hospital, Peking University Cancer Hospital Yunnan, Kunming, Yunnan, China

**Keywords:** breast neoplasms, neoadjuvant chemotherapy, shear wave elastography, stiff rim sign, treatment response

## Abstract

**Background:**

Accurate evaluation of neoadjuvant chemotherapy (NAC) efficacy is critical for optimizing breast cancer treatment. This study investigates shear wave elastography (SWE) for quantifying stiffness changes within lesions and peri-lesional tissues (1–3 mm) and correlating these changes with pathological response to NAC.

**Methods:**

In a prospective cohort of 66 breast cancer patients, SWE parameters (Emax, Emean, Emin, Esd) were measured within tumors and surrounding tissues (1/2/3 mm) pre- and post-NAC. Pathological response was classified via Miller-Payne grading (G3-G5: effective; G1-G2: ineffective). Diagnostic performance was assessed using ROC curves.

**Results:**

Post-NAC, the effective group exhibited significant reductions in Emax, Emean, and Esd within lesions and peri-lesional regions (all *P* < 0.05), while the ineffective group maintained high stiffness. ΔEmax showed high apparent discrimination of pathological response: Intralesional ΔEmax: AUC = 0.941 (95% CI: 0.887-0.995), sensitivity 88.89%, specificity 85.71% (cutoff >49.12%). Peri-lesional ΔEmax (1–3 mm): AUC > 0.9, specificity >90%. The stiff rim sign disappeared exclusively in the effective group post-NAC.

**Conclusion:**

SWE parameters (especially ΔEmax) and dynamic changes in the stiff rim sign are promising non-invasive biomarkers that correlate with pathological response to NAC. These findings support the potential role of SWE in post-treatment response evaluation, though prospective validation in larger cohorts is warranted before clinical implementation.

## 1 Introduction

Breast cancer has now become the most prevalent type of cancer worldwide and remains one of the leading causes of cancer-related deaths among women (1). Neoadjuvant chemotherapy (NAC), an essential treatment modality for breast cancer, refers to systemic chemotherapy administered prior to local therapeutic interventions such as surgery or radiotherapy. NAC effectively eliminates subclinical metastatic lesions in the early stages, leading to a reduction in tumor staging. In turn, creates surgical opportunities for patients and significantly improves their postoperative quality of life. Additionally, NAC provides valuable information regarding *in-vivo* drug sensitivity, facilitating accurate assessment of patients’ responsiveness to chemotherapy agents (2, 3). In recent years, with the continuous optimization of NAC treatment regimens and the precise application of chemotherapy drugs, increasingly more breast cancer patients have benefited from this approach. It is worth mentioning that the achievement of pathological complete response (pCR) after NAC not only indicates a favorable prognosis for patients but also serves as a critical metric for evaluating treatment efficacy. However, it cannot be ignored that a subset of patients still exhibits poor response to NAC treatment, and some even experience disease progression. Therefore, accurate and timely efficacy evaluation during NAC treatment is particularly crucial. Through such assessment, patients who do not respond to initial treatment or are unlikely to benefit can be identified in a timely manner, allowing for early adjustment of treatment plans and optimization of therapeutic outcomes (4, 5).

Currently, evaluating the efficacy of NAC involves three main aspects: clinical assessment, pathological assessment, and imaging assessment. Clinical assessment relies mainly on palpation to compare changes in tumor size before and after NAC to determine its effectiveness. However, this method has a degree of subjective dependence, and its objectivity and accuracy must be improved. Pathological assessment is considered the gold standard for tumor response due to its high accuracy, but it can only be concluded after surgical tissue removal. Therefore, it cannot provide timely evidence for evaluating NAC efficacy.

On the other hand, imaging assessment is NAC’s foundation and implementation guarantee. Doctors and patients welcome it for its non-invasive, convenient, and repeatable advantages. Commonly used imaging methods for NAC evaluation include X-ray examination, magnetic resonance imaging (MRI), positron emission tomography/computed tomography (PET/CT), and ultrasonography (US). For some lesions with unclear boundaries after NAC or inherently indistinct boundaries (such as non-mass breast cancer), as well as lesions with dense breast glands, X-ray examination often yields inaccurate measurements and provides limited information regarding axillary lymph node status (6). MRI, as a non-radiative and non-invasive examination method, can effectively distinguish between residual lesions and fibrosis after chemotherapy, demonstrating high effectiveness. However, its high cost and long examination time restrict its repeated application in NAC patients. Although PET/CT has specific diagnostic value, it is still widely used in clinical practice due to its radioactivity and high cost.

In contrast, US allows for the evaluation of lesion changes and the detection of axillary lymph node responses. Its simplicity, affordability, non-radiation, and repeatability make the US essential during breast cancer NAC and have gained widespread application. Some studies suggest that the US is equal to MRI in terms of accuracy for evaluating residual lesions after NAC (7).

As an emerging technology, ultrasound elastography (UE) enables qualitative or quantitative measurement of tissue stiffness. UE has been widely applied in the medical field, and its significant advantages are that it is non-invasive, simple, and reproducible. Among various UE techniques, shear wave elastography (SWE) stands out due to its precise measurement of the propagation speed of shear waves in tissues, thereby obtaining Young’s modulus value. This critical parameter comprehensively reflects tissues’ maximum, average, and minimum stiffness values, providing detailed information about tissue hardness. Numerous studies have indicated that significant changes occur in the extracellular matrix during cancer progression, consisting of connective fibrous tissue, which makes the tissue texture denser and harder (8). Changes in tumor stiffness during NAC may reflect treatment-related alterations in tumor tissue. In a prospective study, SWE parameters measured before, during, and after NAC differed between patients with and without pathological response (9). Indeed, several studies have confirmed SWE’s effectiveness in evaluating NAC’s efficacy for breast cancer (10–12). Malignant tumors possess unique biological characteristics, such as expansive growth and breaking through the extracellular matrix, allowing cancer cells to infiltrate and spread to surrounding tissues, increasing stiffness. Some studies have further pointed out that the stiffness of the surrounding tissue of breast malignant lesions is often higher than that of the internal tissue (13). Based on this finding, this study uses SWE technology to quantitatively measure changes in Young’s modulus values within breast cancer lesions and their surrounding tissues (including 1mm, 2mm, and 3mm ranges) before and after NAC treatment. This approach will provide further insight into the practical application value of SWE in evaluating the efficacy of NAC for breast cancer.

However, it should be noted that SWE measurements in this study were performed before NAC and after completion of all chemotherapy cycles, rather than at interim time points. Therefore, this study aims to validate SWE-derived stiffness changes as biomarkers correlating with final pathological response, rather than as an early predictive tool for mid-treatment decision-making. The findings are intended to establish a foundation for future longitudinal studies with serial SWE assessments during early treatment cycles.

## 2 Materials and methods

### 2.1 Study population

This study was conducted as a prospective investigation. The study sample consisted of 66 breast cancer patients who underwent NAC at the Third Affiliated Hospital of Kunming Medical University between February 2022 and February 2023, involving a total of 66 breast lesions (**Figure 1**).

**Figure 1:**
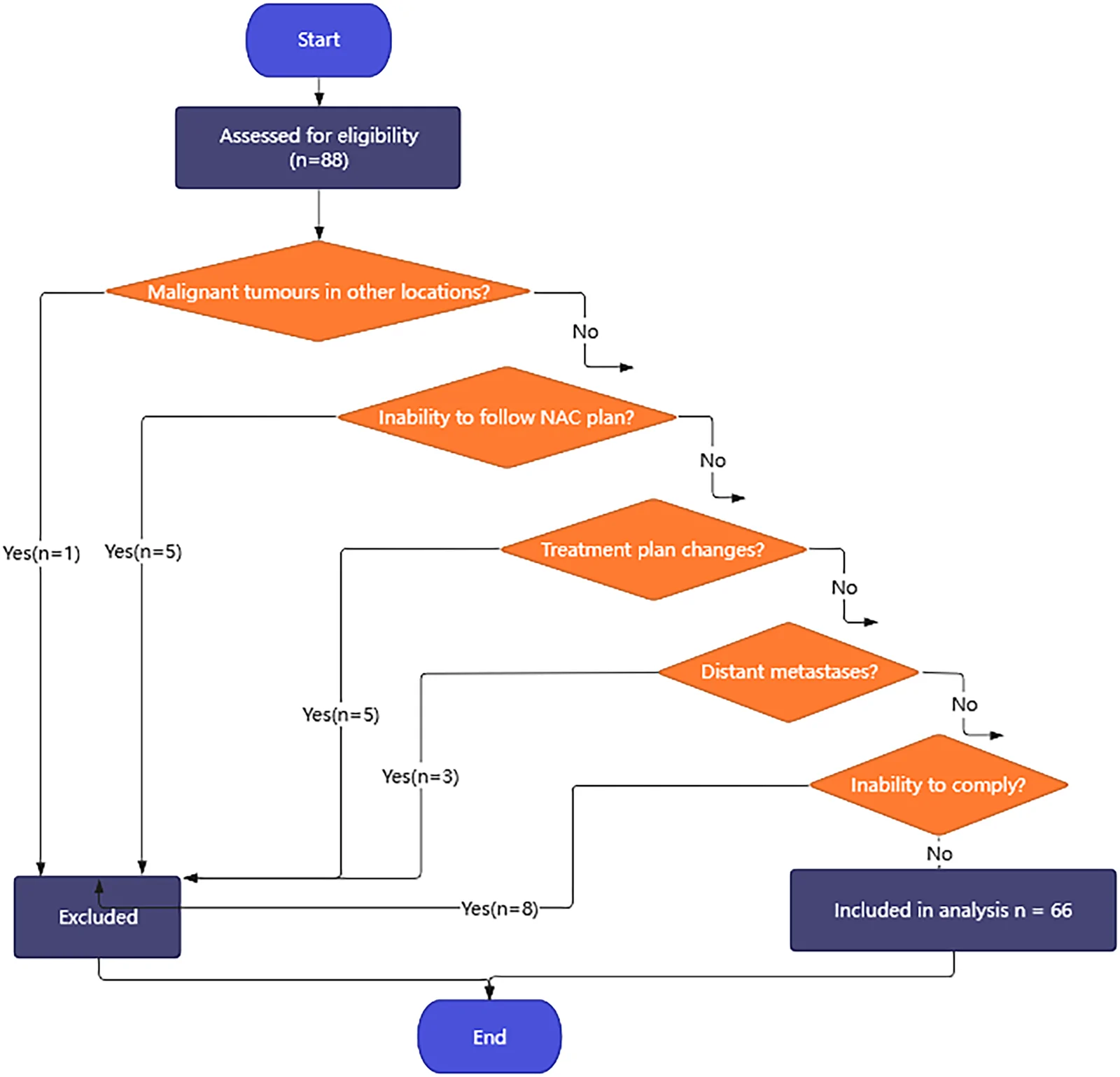
Flow diagram of patient enrollment. A total of 88 patients with breast cancer were assessed for eligibility. 22 patients were excluded for the following reasons: malignant tumors in other locations (n=1), inability to follow the NAC treatment plan (n=5), changes in the treatment plan during chemotherapy (n=5), development of distant metastases (n=3), and inability to comply with all study procedures (n=8). Finally, 66 patients were included in the analysis.

The inclusion criteria were as follows: (1) Diagnosed with breast cancer through histopathological examination of core needle biopsy before chemotherapy; (2) Met the treatment indications for NAC; (3) Underwent routine US and SWE before and after the final chemotherapy session; (4) Completed the entire course of chemotherapy and ultimately underwent surgical treatment; (5) Had no history of previous local or systemic chemotherapy.

Exclusion criteria included: (1) Presence of malignant tumors in other locations; (2) Inability to follow the NAC treatment plan; (3) Changes in the treatment plan during chemotherapy; (4) Development of distant metastases; (5) Inability to comply with all procedures required for this study.

The study was approved by the Ethics Committee of the Third Affiliated Hospital of Kunming Medical University, and all patients were ensured to have signed informed consent forms.

### 2.2 Instrumentation and methods

This study used the Mindray Resona 7 color Doppler ultrasound diagnostic instrument equipped with a linear-array transducer operating at a frequency of 5–14 MHz. Patients were positioned in the supine position with adequate exposure of the breast. Conventional B-mode US was first performed to record the lesion location and to measure the maximum lesion diameter.

Subsequently, SWE was performed for elasticity assessment of the lesion. During the SWE examination, patients were instructed to hold their breath, and the probe was placed steadily over the lesion without applying additional external pressure. The SWE mode was then activated to acquire elastographic images. Grayscale images and SWE images were displayed simultaneously in a split-screen mode, with the color-coded SWE map overlaid on the corresponding grayscale image. The SWE images were color-coded, ranging from blue (indicating softer tissue) to red (indicating stiffer tissue).

A region of interest (ROI) was selected to include the lesion and a portion of the surrounding normal glandular or fatty tissue. Quality control criteria required a uniformly green background on the quality map without purple artifacts, indicating reliable shear wave propagation and adequate image quality (**Figure 2A**). The Young’s modulus display scale was set with an upper threshold of 140 kPa. The system automatically calculated quantitative elasticity values within the ROI, expressed as Young’s modulus in kilopascals (kPa).

**Figure 2:**
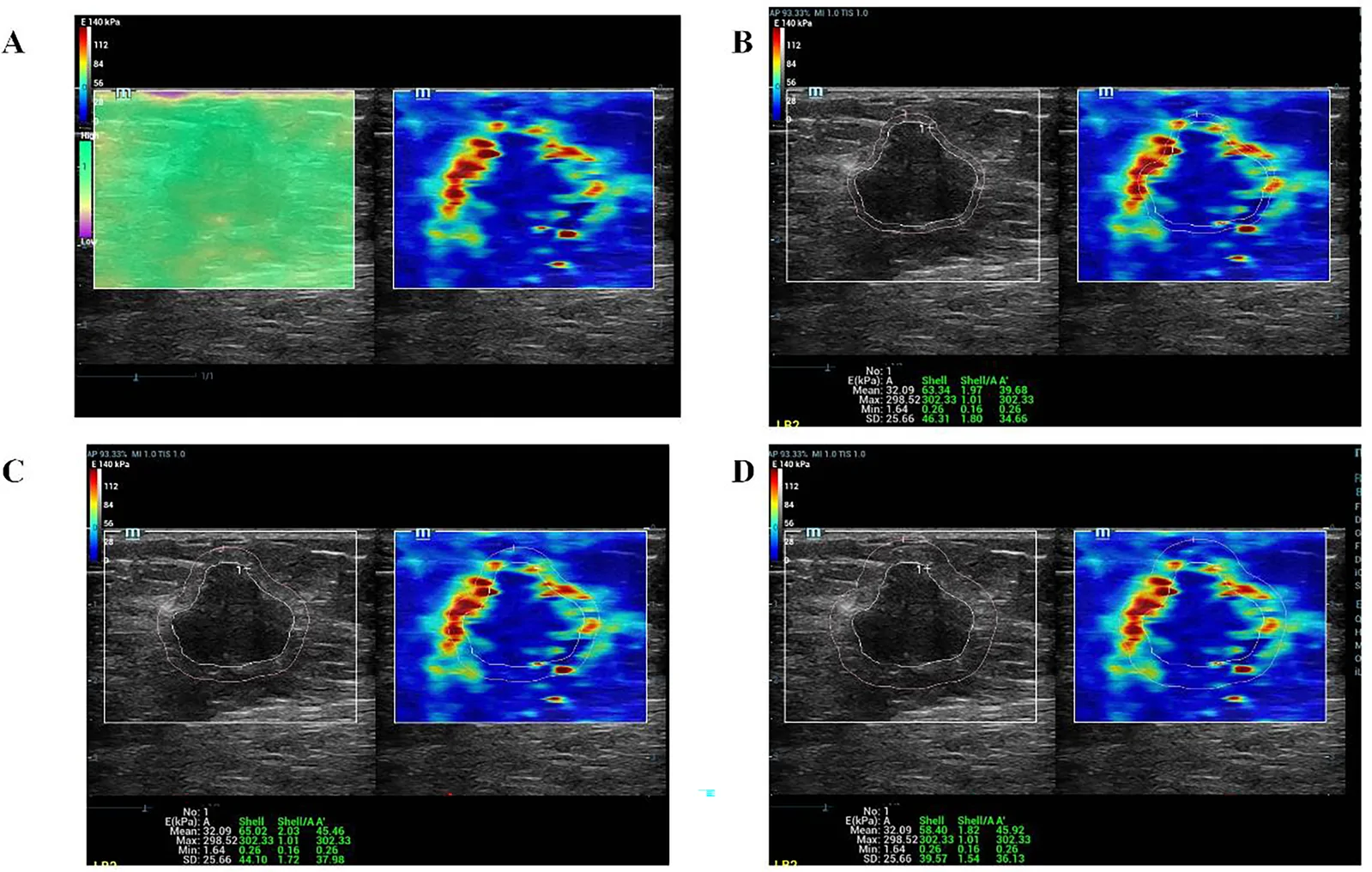
Representative SWE quality control, stiff rim sign, and peri-lesional shell measurements. **(A)** Representative SWE images illustrating quality control assessment on the left and the presence of the stiff rim sign on the right. The SWE color map is overlaid on the corresponding grayscale image. A uniform green background on the quality map without purple artifacts indicates reliable shear wave propagation and acceptable image quality. A circumferential red-orange high-stiffness area surrounding the lesion margin is identified as the stiff rim sign. **(B–D)** Peri-lesional shell-based SWE measurements generated using the system’s built-in Shell function after lesion boundary delineation with the tracing method. Concentric ring-shaped regions were automatically created outward from the lesion margin. B shows elasticity measurements within the 1-mm peri-lesional shell, C within the 2-mm shell, and D within the 3-mm shell. For each shell, quantitative elasticity parameters including Emax, Emean, Emin, and Esd were automatically calculated.

Lesion boundaries were delineated on the grayscale image using the tracing method, after which the system automatically measured intralesional elasticity parameters, including the maximum elasticity value (Emax), mean elasticity value (Emean), minimum elasticity value (Emin), and the standard deviation of elasticity (Esd).

For peri-lesional elasticity assessment, the built-in Shell function of the ultrasound system was applied. After delineation of the lesion boundary using the tracing method, concentric peri-lesional shells were automatically generated outward from the lesion margin. The shell thickness was sequentially adjusted to 1 mm, 2 mm, and 3 mm to obtain ring-shaped regions surrounding the lesion. At each shell distance, elasticity parameters including Emax, Emean, Emin, and Esd were measured and recorded. Specifically, measurements were first obtained with the shell set to 1 mm (**Figure 2B**), then repeated with the shell adjusted to 2 mm (**Figure 2C**), and finally with the shell adjusted to 3 mm (**Figure 2D**).

SWE measurements were performed both before initiation of NAC (pre-NAC) and after completion of the final chemotherapy cycle (post-NAC). The percentage change in elasticity parameters was calculated to evaluate treatment-related stiffness variation. The change rate of Emax (ΔEmax) was defined as: ΔEmax = [Emax(pre-NAC) − Emax(post-NAC)]/Emax(pre-NAC) × 100%. The same formula was applied to calculate ΔEmean and ΔEsd, as well as the corresponding change rates for peri-lesional regions at 1 mm, 2 mm, and 3 mm from the lesion boundary.

All ultrasonographic and SWE examinations were performed by two physicians, each with more than five years of experience in breast US. Both physicians underwent standardized SWE operation training prior to the study, with unified protocols for image acquisition and ROI placement.

### 2.3 Chemotherapy regimen

According to the *Chinese Anti-Cancer Association Guidelines and Standards for the Diagnosis and Treatment of Breast Cancer (2021 Edition)* (14), all patients received NAC regimens consisting of anthracycline and/or taxane-based regimens. Each cycle was administered every 21 days, for a total of 6–8 cycles.

### 2.4 Histopathological evaluation

In assessing the efficacy of NAC for breast cancer, this study adopted the histopathological grading system proposed by Miller and Payne. This system compares the pathological changes between the surgically removed specimens after chemotherapy and the biopsy tissues before chemotherapy, classifying the treatment response into five grades. The specific grading criteria are as follows: Grade 1 (G1): No significant change in tumor cells or only minor changes in individual cells, with no overall reduction in cellularity. Grade 2 (G2): A noticeable reduction in the number of tumor cells, but the reduction proportion is less than 30%. Grade 3 (G3): Significant reduction in tumor cells, with an estimated reduction proportion between 30% and 90%. Grade 4 (G4): Only scattered infiltrating tumor cells remain, with an estimated reduction proportion exceeding 90%. Grade 5 (G5): No residual infiltrating cancer cells are visible.

Based on the above grading evaluation criteria, patients with G3 to G5 grades were classified as the effective group, while those with G1 to G2 grades were classified as the ineffective group. This classification method facilitates a more accurate evaluation of the chemotherapy regimen’s efficacy and provides valuable insights for subsequent treatment.

### 2.5 Statistical analysis

This study used SPSS 21.0 and MedCalc 15.2 statistical software for in-depth data analysis. The age of patients, lesion size, and elasticity values (including Emax, Emean, Emin, and Esd) within and at 1mm, 2mm, and 3mm peripheries of the lesions were tested and found to conform to a normal distribution. These variables were described using mean ± standard deviation (x ± s). To compare SWE parameters between the effective and ineffective groups, an independent samples t-test was employed. For comparisons involving multiple sample means, one-way analysis of variance (ANOVA) was applied.

Additionally, count data were presented as frequencies (percentages), and comparisons between two groups were performed using the χ2 test or Fisher’s exact test. Receiver operating characteristic (ROC) curves were constructed to evaluate SWE’s performance in predicting NAC’s efficacy for breast cancer. The area under the curve (AUC), cutoff values, sensitivity, and specificity of each parameter were calculated. A *P*-value less than 0.05 was considered statistically significant. To account for multiple testing in the between-group comparisons of percentage changes, the Benjamini-Hochberg false discovery rate (FDR) procedure was applied to the 12 comparisons of ΔEmax, ΔEmean, and ΔEsd across the intralesional and 1-, 2-, and 3-mm peri-lesional locations, treated as one family of tests. Both unadjusted P values and FDR-adjusted *q* values are reported in **Tables 1** and **2**; *q* < 0.05 was considered statistically significant for these comparisons. Other analyses, including comparisons of absolute SWE values before or after NAC, are reported with unadjusted *P* values and interpreted as exploratory.

**Table 1:** Comparison of change rates in SWE parameters within lesions after NAC (%).

Parameter	Effective group (n=45)	Ineffective group (n=21)	t value	*P* value	*q*-values
ΔEmax	67.86 ± 14.62	21.28 ± 27.16	7.38	<0.001	<0.001
ΔEmean	46.55 ± 24.11	25.45 ± 31.88	2.98	0.004	0.004
ΔEsd	64.07 ± 20.31	25.97 ± 26.03	6.48	<0.001	<0.001

Δ= (Pre-NAC value−Post-NAC value)/Pre-NAC value×100%.

ΔEmax and ΔEsd demonstrated most significant treatment response discrimination. ΔEmin was excluded from this analysis because percentage change was uninterpretable due to baseline values approaching zero. FDR-adjusted q-values were calculated using the Benjamini-Hochberg procedure. All significant comparisons remained statistically significant after FDR correction (*q* < 0.05). The *q* values were adjusted together across the 12 percentage-change comparisons reported in **Tables 1** and **2**.

**Table 2:** Comparison of change rates in peri-lesional SWE parameters at 1mm, 2mm, and 3mm after NAC (%).

Parameter	Effective group (n=45)	Ineffective group (n=21)	t value	*P* value	*q*-values
ΔEmax-1mm	66.94 ± 14.77	26.50 ± 33.69	5.27	<0.001	<0.001
ΔEmean-1mm	52.51 ± 17.12	27.36 ± 18.78	5.39	<0.001	<0.001
ΔEsd-1mm	64.90 ± 17.14	23.94 ± 39.87	4.52	<0.001	<0.001
ΔEmax-2mm	67.76 ± 14.72	32.29 ± 25.95	5.84	<0.001	<0.001
ΔEmean-2mm	52.19 ± 17.57	27.92 ± 18.14	5.17	<0.001	<0.001
ΔEsd-2mm	63.81 ± 16.75	26.12 ± 32.00	5.08	<0.001	<0.001
ΔEmax-3mm	66.97 ± 14.20	33.09 ± 25.44	5.70	<0.001	<0.001
ΔEmean-3mm	51.71 ± 17.51	28.34 ± 18.50	4.96	<0.001	<0.001
ΔEsd-3mm	62.78 ± 16.91	26.64 ± 29.19	6.36	<0.001	<0.001

Δ= (Pre-NAC value−Post-NAC value)/Pre-NAC value×100%.

ΔEmax, ΔEmean, and ΔEsd demonstrated significant treatment response discrimination (*P* < 0.001). ΔEmin was excluded from this analysis because percentage change was uninterpretable due to baseline values approaching zero. FDR-adjusted q-values were calculated using the Benjamini-Hochberg procedure. All significant comparisons remained statistically significant after FDR correction (*q* < 0.05). The *q* values were adjusted together across the 12 percentage-change comparisons reported in **Tables 1** and **2**.

Normality of continuous variables was assessed using the Shapiro-Wilk test. For parameters with non-normal distribution, between-group comparisons were performed using the Mann-Whitney U test, and paired comparisons (pre- vs. post-NAC) were performed using the Wilcoxon signed-rank test. Pairwise comparisons of ROC curves were performed using DeLong’s test to evaluate whether the AUCs of different parameters differed significantly.

The reported analyses included 66 patients (45 in the effective group and 21 in the ineffective group). No missing values were reported for the primary SWE parameters, and no data imputation was performed. Because low baseline Emin values produced unstable percentage changes, ΔEmin was omitted from the percentage-change comparisons and ROC analyses; absolute Emin measurements were retained. No fixed statistical cutoff was used in the revised manuscript to justify excluding individual SWE observations. Inter-observer agreement for quantitative SWE parameters was assessed at each measurement location and time point using single-measure, absolute-agreement intraclass correlation coefficients (ICCs) with 95% confidence intervals.

## 3 Results

### 3.1 Overview of essential patient information

All 66 patients (corresponding to 66 breast lesions) included in this study were diagnosed with breast cancer through core needle biopsy and histopathological examination before chemotherapy. The essential characteristics of the patients are summarized in **Table 3**. For the group of patients with effective chemotherapy response (effective group), the median age was 46.67 years, with a standard deviation of 10.49 years, ranging from 21 to 74 years. In contrast, the median age of the group with ineffective chemotherapy response (ineffective group) was 49.76 years, with a standard deviation of 6.60 years, ranging from 36 to 64 years.

**Table 3:** Comparison of baseline patient characteristics.

Features	Effective group (n=45)	Ineffective group (n=21)	t/χ² value	*P* value
Age (years)	46.67 ± 10.49	49.76 ± 6.60	-1.46	0.151
Maximum diameter (mm)	42.71 ± 21.59	48.33 ± 21.26	-0.99	0.326
Molecular subtype (n)			5.05	0.08
Luminal	27	18		
HER2+	12	1		
TNBC	6	2		
Ki67 index (n)			2.11	0.146
≤20%	12	7	0.31*	0.577*
>20%	33	14		

*Data presented as mean ± standard deviation or number of cases (n).

χ² test for categorical variables, independent t-test for continuous variables.

Fisher’s exact test for Ki67 subgroup comparison.

Regarding lesion size, the mean maximum diameter of lesions in the effective group was 42.71 mm, with a standard deviation of 21.59 mm, ranging from 8 mm to 87 mm. The mean maximum diameter of lesions in the ineffective group was slightly larger, at 48.33 mm, with a standard deviation of 21.26 mm, ranging from 10 mm to 100 mm. The pathological response after NAC was evaluated through postoperative specimen analysis, using the Miller and Payne (MP) grading system as a reference. Among the 66 patients, 45 (68.18%) were classified as the effective group (Miller-Payne G3-G5), while 21 (31.82%) were classified as the ineffective group (G1-G2).

### 3.2 Comparative analysis of SWE parameters in lesions before NAC

Before NAC, we compared SWE parameters within the lesions between the effective and ineffective groups. The results showed that the maximum elasticity value (Emax) within the lesions of the effective group was 225.27 ± 80.70 Kpa, while that of the ineffective group was 246.54 ± 96.29 Kpa. Statistical analysis revealed no significant difference in Emax between the two groups. Similarly, we compared the Emean, Emin, and Esd. We found no statistically significant differences in these parameters between the effective and ineffective groups (*P*>0.05). Detailed data are presented in **Table 4**.

**Table 4:** Comparison of SWE parameters within lesions before NAC (kPa).

Parameter	Effective group (n=45)	Ineffective group (n=21)	t value	*P* value
Emax	225.27 ± 80.70	246.54 ± 96.29	-0.94	0.352
Emean	60.67 ± 17.94	62.96 ± 28.27	-0.40	0.691
Emin	10.00 ± 9.18	8.93 ± 6.28	0.48	0.632
Esd	32.42 ± 10.17	37.35 ± 18.59	-1.39	0.169

SWE, shear wave elastography; NAC, neoadjuvant chemotherapy; Emax, maximum elasticity value; Emean, mean elasticity value; Emin, minimum elasticity value; Esd, standard deviation of elasticity.

Data presented as mean ± standard deviation. Comparisons by independent t-test.

This study compared SWE parameters at 1mm, 2mm, and 3mm around the lesions between the effective and ineffective groups before NAC. The results indicated that within the 1mm perimeter of the lesions, the Emax of the effective group was 216.02 ± 61.05 Kpa, compared to 228.57 ± 82.83 Kpa in the ineffective group, with no statistically significant difference between the two groups (*P*>0.05). Likewise, comparisons of Emean, Emin, and Esd within this perimeter showed no statistically significant differences between the effective and ineffective groups (*P*>0.05). Specifically, the Emean values were 65.43 ± 13.48 Kpa and 64.37 ± 15.92 Kpa, Emin values were 10.78 ± 7.55 Kpa and 8.61 ± 5.21 Kpa, and Esd values were 37.03 ± 10.62 Kpa and 41.29 ± 18.99 Kpa for the effective and ineffective groups, respectively. Additionally, no statistically significant differences were found in the Emax, Emean, Emin, and Esd parameters at 2mm and 3mm perimeters around the lesions between the effective and ineffective groups (*P*>0.05). Detailed data are provided in **Table 5**.

**Table 5:** Comparison of peri-lesional SWE parameters at 1mm, 2mm, and 3mm before NAC (kPa).

Parameter	Effective group (n=45)	Ineffective group (n=21)	t value	*P* value
Emax-1mm	216.02 ± 61.05	228.57 ± 82.83	-0.69	0.491
Emean-1mm	65.43 ± 13.48	64.37 ± 15.92	0.28	0.780
Emin-1mm	10.78 ± 7.55	8.61 ± 5.21	1.19	0.239
Esd-1mm	37.03 ± 10.62	41.29 ± 18.99	-1.17	0.247
Emax-2mm	227.71 ± 67.62	247.70 ± 80.52	-1.05	0.297
Emean-2mm	61.94 ± 12.77	61.61 ± 14.44	0.09	0.925
Emin-2mm	8.55 ± 6.24	7.31 ± 5.16	0.79	0.433
Esd-2mm	35.18 ± 9.18	39.81 ± 17.30	-1.42	0.160
Emax-3mm	231.42 ± 70.66	255.03 ± 82.96	-1.20	0.236
Emean-3mm	58.24 ± 12.25	58.86 ± 13.14	-0.19	0.852
Emin-3mm	6.82 ± 4.89	5.87 ± 4.88	0.73	0.465
Esd-3mm	34.08 ± 9.24	38.46 ± 15.70	-1.42	0.159

Suffixes (-1mm, -2mm, -3mm) denote measurement distance from lesion boundary.

All values presented as mean ± standard deviation.

### 3.3 Comparative analysis of SWE parameters within lesions after NAC

After administering NAC to patients, we compared SWE parameters within the lesions between the effective and ineffective groups. The results revealed a significant decrease in the maximum elasticity value (Emax) within the lesions of the effective group to 65.85 ± 25.92 Kpa. In contrast, it remained higher in the ineffective group, specifically 178.77 ± 62.87 Kpa. The difference in Emax between the two groups was statistically significant (*P* < 0.05). Similarly, we compared the Emean, Emin, and Esd within the lesions. We also found significant differences between the effective and ineffective groups in these parameters, all statistically significant (*P* < 0.05). Detailed data are presented in **Table 6**. These findings suggest that SWE parameters have important reference values when evaluating the efficacy of NAC.

**Table 6:** Comparison of SWE parameters within lesions after NAC (kPa).

Parameter	Effective group (n=45)	Ineffective group (n=21)	t value	*P* value
Emax	65.85 ± 25.92	178.77 ± 62.87	-7.92	<0.001
Emean	29.90 ± 10.90	42.68 ± 17.29	-3.11	0.004
Emin	11.34 ± 5.32	7.63 ± 5.35	2.64	0.010
Esd	10.47 ± 4.36	25.10 ± 8.57	-7.39	<0.001

All values significant at *P* < 0.05. Data presented as mean ± standard deviation.

Statistical comparisons by independent t-test. Note significant post-NAC stiffness reduction in effective group.

After NAC treatment, this study compared SWE parameters in the surrounding tissues of lesions between the effective and ineffective groups. The results showed that within the 1mm perimeter of the lesions, the Emax (maximum elasticity value) of the effective group was 67.08 ± 26.87 Kpa, significantly lower than the 154.07 ± 55.82 Kpa of the ineffective group, and the difference between the two groups was statistically significant (*P* < 0.05). Likewise, in terms of Emean within this perimeter, the effective group had a value of 29.90 ± 9.03 Kpa, compared to 45.30 ± 11.39 Kpa in the ineffective group, with a significant difference (*P* < 0.05). For Emin, the effective group was higher at 10.08 ± 3.35 Kpa compared to 7.54 ± 5.08 Kpa in the ineffective group, and the difference was statistically significant (*P* < 0.05). Additionally, in terms of Esd, the effective group was significantly lower at 12.14 ± 5.36 Kpa compared to 27.25 ± 8.56 Kpa in the ineffective group (*P* < 0.05). It is worth noting that these significant differences were not only observed within the 1mm perimeter of the lesions but also extended to the 2mm and 3mm perimeters, involving all SWE parameters, including Emax, Emean, Emin, and Esd (*P* < 0.05). Detailed data are provided in **Table 7**. These findings suggest that post-NAC SWE parameters may serve as valuable references for evaluating treatment efficacy.

**Table 7:** Comparison of peri-lesional SWE parameters at 1mm, 2mm, and 3mm After NAC (kPa).

Parameter	Effective group (n=45)	Ineffective group (n=21)	t value	*P* value
Emax-1mm	67.08 ± 26.87	154.07 ± 55.82	-6.78	<0.001
Emean-1mm	29.90 ± 9.03	45.30 ± 11.39	-5.93	<0.001
Emin-1mm	10.08 ± 3.35	7.54 ± 5.08	2.43	0.018
Esd-1mm	12.14 ± 5.36	27.25 ± 8.56	-7.44	<0.001
Emax-2mm	68.54 ± 27.30	158.49 ± 56.94	-6.88	<0.001
Emean-2mm	28.49 ± 8.60	43.45 ± 11.80	-5.20	<0.001
Emin-2mm	8.37 ± 2.97	6.08 ± 4.00	2.60	0.011
Esd-2mm	12.11 ± 5.38	26.33 ± 7.90	-8.57	<0.001
Emax-3mm	71.26 ± 26.50	161.23 ± 58.49	-6.73	<0.001
Emean-3mm	27.02 ± 8.12	41.47 ± 11.81	-5.08	<0.001
Emin-3mm	7.30 ± 2.88	5.33 ± 3.87	2.30	0.024
Esd-3mm	12.01 ± 5.05	25.56 ± 7.59	-8.60	<0.001

Suffixes denote measurement distance from lesion boundary. All *P* < 0.05 except where noted. Note progressive stiffness reduction extending to 3mm periphery in effective group.

### 3.4 Comparison of the change rates of SWE parameters in lesions before and after NAC

In this study, we further analyzed the change rates of SWE parameters within lesions before and after NAC. The results showed that ΔEmax within lesions in the effective group reached 67.86 ± 14.62% after NAC treatment, while the change rate in the ineffective group was only 21.28 ± 27.16%. The difference between the two groups was statistically significant (*P* < 0.05). Similarly, when comparing the change rates of Emean (ΔEmean) and Esd (ΔEsd) within lesions before and after NAC, significant differences were also observed between the effective and ineffective groups, and these differences were statistically significant (*P* < 0.05). In contrast, the percentage change of Emin (ΔEmin) yielded extreme and uninterpretable values due to very low baseline Emin values (frequently < 10 kPa), which result in division by near-zero denominators. Therefore, ΔEmin was excluded from further analysis. These findings provide substantial evidence for a deeper understanding of the effects of NAC on SWE parameters within lesions. See **Table 1** for details.

This study conducted a detailed comparative analysis of the SWE change rates parameters in the perilesional tissue at 1mm, 2mm, and 3mm before and after NAC. The results showed that within the 1mm perimeter of the lesion, ΔEmax in the effective group was 66.94 ± 14.77%, significantly higher than that in the ineffective group (26.50 ± 33.69%). The difference between the two groups was statistically significant (*P* < 0.05). Similarly, in terms of the change rates of Emean (ΔEmean) and Esd (ΔEsd) within this range, the effective group was also significantly higher than the ineffective group, and the differences were statistically significant (*P* < 0.05). Specifically, the change rate of Emean in the effective group was 52.51 ± 17.12%, while that in the ineffective group was 27.36 ± 18.78%; the change rate of Esd in the effective group was 64.90 ± 17.14%, while that in the ineffective group was 23.94 ± 39.87%. ΔEmin was not analyzed due to the reasons described above. It is worth noting that these significant differences not only existed within the 1mm perimeter of the lesion but were also observed within the 2mm and 3mm perimeters of the lesion, involving SWE parameters such as the change rates of Emax, Emean, and Esd (*P* < 0.05). Detailed data are presented in **Table 2**. These findings provide strong evidence to support the evaluation of the effects of NAC on the elastic characteristics of perilesional tissue.

### 3.5 Observation of SWE and “stiff rim” changes before and after NAC treatment

**Figure 3** shows a representative case from the effective group in which the stiff rim sign disappeared after NAC, accompanied by a reduction in lesion stiffness. **Figure 4** shows a representative case from the ineffective group in which the sign persisted after NAC. Across the cohort, the stiff rim sign disappeared in 32 of 45 patients (71.1%) in the effective group and persisted in the remaining 13 patients (28.9%). It persisted in all 21 patients (100%) in the ineffective group. When persistence of the stiff rim sign after NAC was used to identify ineffective pathological response, sensitivity was 100% (95% CI: 83.9%-100%) and specificity was 71.1% (95% CI: 56.0%-83.0%).

**Figure 3:**
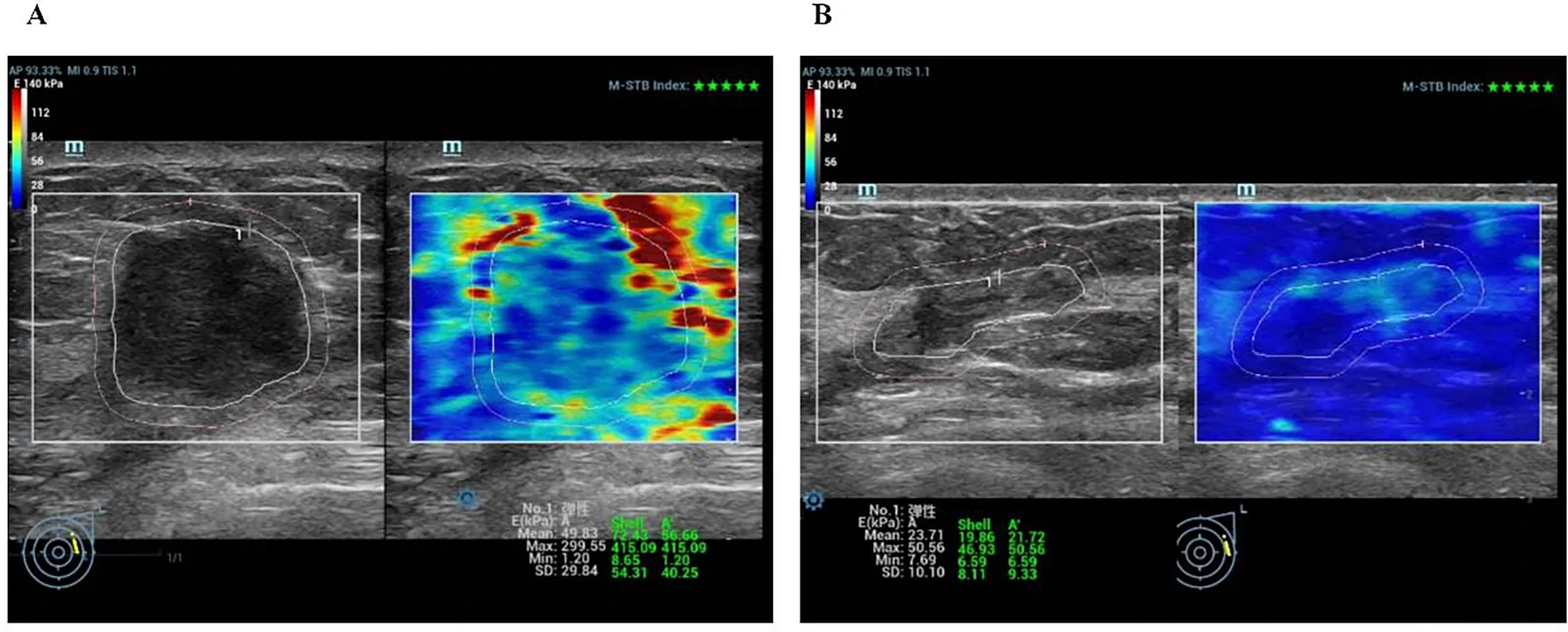
Changes in shear wave elastography features before and after neoadjuvant chemotherapy in the effective group. **(A)** The pre-NAC SWE image, characterized by high intralesional stiffness (Emax = 299.55 kPa) and the presence of a circumferential peri-lesional high-stiffness area, consistent with the stiff rim sign. **(B)** The post-NAC SWE image, in which a marked reduction in lesion stiffness is observed (Emax = 50.56 kPa), accompanied by complete disappearance of the stiff rim sign. These imaging changes reflect significant alterations in the elastic properties of the breast cancer lesion and surrounding tissue following effective neoadjuvant chemotherapy.

**Figure 4:**
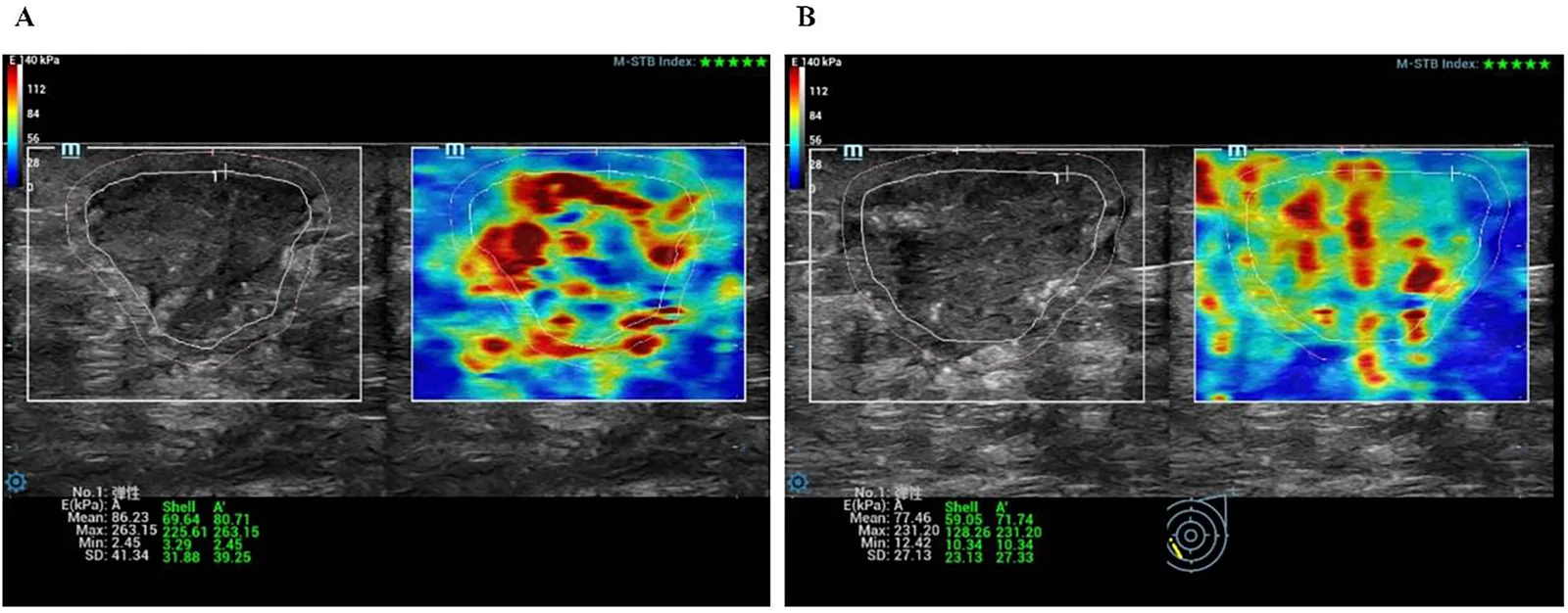
Persistent shear wave elastography features before and after neoadjuvant chemotherapy in the ineffective group. **(A)** The pre-NAC SWE image, characterized by high intralesional stiffness (Emax = 263.15 kPa) and a prominent circumferential peri-lesional high-stiffness area, consistent with the stiff rim sign. **(B)** The post-NAC SWE image, in which the lesion continues to exhibit high stiffness (Emax = 231.20 kPa), with persistence of the stiff rim sign. These findings indicate that neoadjuvant chemotherapy did not result in a significant reduction in tissue stiffness or alteration of peri-lesional elastic characteristics in the ineffective group.

Quantitative assessment of the stiff rim sign revealed that it disappeared in 32 of 45 patients (71.1%) in the effective group, while it persisted in 13 patients (28.9%). In the ineffective group, the stiff rim sign persisted in all 21 patients (100%), with no cases showing disappearance. Using the presence of a persistent stiff rim sign after NAC as a predictor of ineffective pathological response yielded a sensitivity of 100% (95% CI: 83.9%-100%) and a specificity of 71.1% (95% CI: 56.0%-83.0%).

### 3.6 Comparative analysis of diagnostic performance of SWE parameter change rates in lesions and surrounding tissues at 1mm, 2mm, and 3mm before and after NAC

This study compared the diagnostic performance of SWE parameter change rates, including ΔEmax, ΔEmean, and ΔEsd, in lesions and surrounding tissues at 1mm, 2mm, and 3mm before and after NAC. The results showed that the AUCs (areas under the curve) of ΔEmax in the lesions and at various distances from the lesions were 0.941 (95% CI: 0.887-0.995), 0.916 (95% CI: 0.850-0.983), 0.910 (95% CI: 0.842-0.978), and 0.905 (95% CI: 0.834-0.976), respectively. The AUCs of ΔEmean were 0.714 (95% CI: 0.581-0.847), 0.853 (95% CI: 0.763-0.943), 0.849 (95% CI: 0.755-0.942), and 0.843 (95% CI: 0.747-0.939), respectively. ΔEsd also demonstrated high diagnostic value with AUCs of 0.883 (95% CI: 0.799-0.966), 0.876 (95% CI: 0.791-0.961), 0.888 (95% CI: 0.806-0.969), and 0.883 (95% CI: 0.800-0.965), respectively. Within the lesions, ΔEmax achieved a sensitivity of 88.89% and a specificity of 85.71%. In the surrounding tissues, the sensitivity and specificity of ΔEmax at 1mm were 80.00% and 90.48%, respectively; at 2mm, they were 77.78% and 90.48%, respectively; and at 3mm, they maintained high levels of 75.56% and 90.48%, respectively. Detailed data can be found in **Table 8** and **Figure 5**. Pairwise comparisons using DeLong’s test showed no statistically significant differences between the AUC of intralesional ΔEmax and those of peri-lesional ΔEmax at 1 mm (AUC difference: 0.025, *P* = 0.646), 2 mm (AUC difference: 0.031, *P* = 0.457), or 3 mm (AUC difference: 0.036, *P* = 0.380), indicating no statistically significant AUC difference was detected in this cohort.

**Table 8:** Diagnostic performance of SWE parameter change rates (ΔEmax, ΔEmean, ΔEsd) for NAC response prediction.

Location	Parameter	Cut-off (%)	Sensitivity (%)	Specificity (%)	AUC (95% CI)
Intralesional					
	ΔEmax	>49.12	88.89	85.71	0.941 (0.887-0.995)
	ΔEmean	>26.63	82.22	57.14	0.714 (0.581-0.847)
	ΔEsd	>54.82	77.78	95.24	0.883 (0.799-0.966)
Peri-lesional 1mm					
	ΔEmax	>55.97	80.00	90.48	0.916 (0.850-0.983)
	ΔEmean	>44.10	75.56	85.71	0.853 (0.763-0.943)
	ΔEsd	>42.52	86.67	76.19	0.876 (0.791-0.961)
Peri-lesional 2mm					
	ΔEmax	>58.23	77.78	90.48	0.910 (0.842-0.978)
	ΔEmean	>42.14	77.78	85.71	0.849 (0.755-0.942)
	ΔEsd	>35.29	93.33	71.43	0.888 (0.806-0.969)
Peri-lesional 3mm					
	ΔEmax	>56.47	75.56	90.48	0.905 (0.834-0.976)
	ΔEmean	>49.49	62.22	95.24	0.843 (0.747-0.939)
	ΔEsd	>34.91	91.11	71.43	0.883 (0.800-0.965)

AUC = area under ROC curve; CI = confidence interval.

Optimal cut-off values determined by Youden index. ΔEmax demonstrated excellent diagnostic performance (AUC >0.9) across all measurement locations.

ΔEmin was excluded from this analysis due to extreme variability resulting from division by near-zero baseline values, rendering percentage changes uninterpretable.

**Figure 5:**
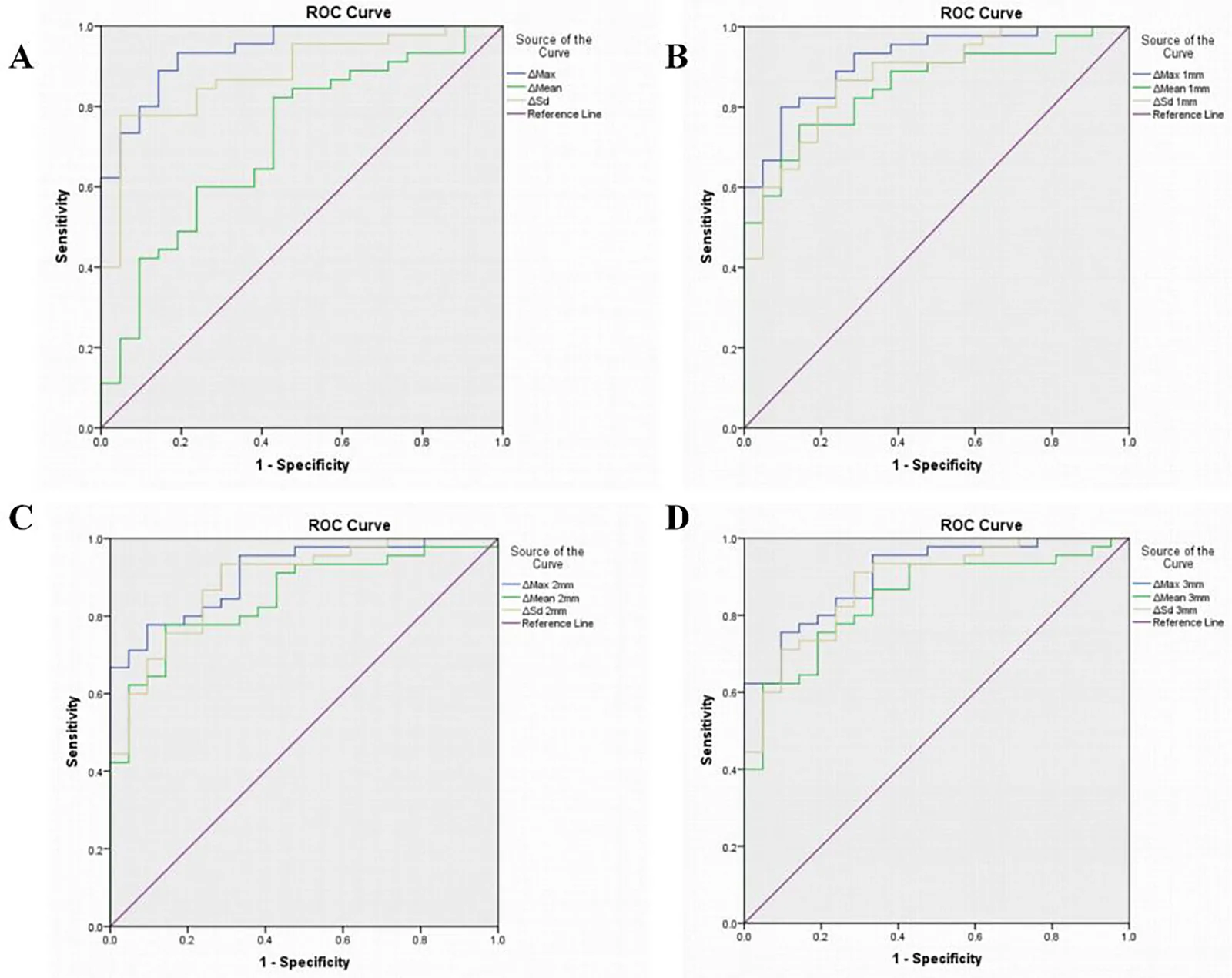
Receiver operating characteristic analysis of SWE parameter changes rates for predicting neoadjuvant chemotherapy response. **(A)** ROC curves for intralesional parameters. **(B–D)** ROC curves for peri-lesional measurements obtained at 1 mm, 2 mm, and 3 mm from the lesion boundary, respectively. The change rates of ΔEmax, ΔEmean, and ΔEsd were evaluated. ΔEmax demonstrated excellent diagnostic performance across both intralesional and peri-lesional regions, with area under the curve (AUC) values exceeding 0.90 at all measurement locations. Sensitivity and specificity values for ΔEmax at each distance are summarized in **Table 8**.

### 3.7 Inter-observer reproducibility

Inter-observer agreement for the quantitative SWE parameters was evaluated using single-measure, absolute-agreement ICCs. As shown in **Table 9**, ICCs across measurement locations and time points ranged from 0.72 to 0.92 for Emax, 0.44 to 0.74 for Emean, 0.26 to 0.51 for Emin, and 0.63 to 0.87 for Esd. Agreement was highest for Emax and lowest for Emin. Intra-observer repeatability could not be assessed because repeat measurements by the same physician were unavailable. Inter-observer agreement for the qualitative stiff rim sign was not assessed.

**Table 9:** Inter-observer reproducibility of SWE parameters.

Time point	Parameter	Location	ICC (95% CI)	*P* value
Pre	Emax	Intralesional	0.91 (0.85-0.95)	<0.001
Pre	Emax	1 mm	0.72 (0.56-0.82)	<0.001
Pre	Emax	2 mm	0.76 (0.61-0.86)	<0.001
Pre	Emax	3 mm	0.77 (0.62-0.86)	<0.001
Post	Emax	Intralesional	0.92 (0.88-0.95)	<0.001
Post	Emax	1 mm	0.81 (0.70-0.88)	<0.001
Post	Emax	2 mm	0.82 (0.71-0.89)	<0.001
Post	Emax	3 mm	0.83 (0.72-0.90)	<0.001
Pre	Emean	Intralesional	0.69 (0.54-0.80)	<0.001
Pre	Emean	1 mm	0.48 (0.27-0.65)	<0.001
Pre	Emean	2 mm	0.45 (0.22-0.63)	<0.001
Pre	Emean	3 mm	0.44 (0.21-0.63)	<0.001
Post	Emean	Intralesional	0.74 (0.60-0.83)	<0.001
Post	Emean	1 mm	0.71 (0.56-0.81)	<0.001
Post	Emean	2 mm	0.72 (0.58-0.82)	<0.001
Post	Emean	3 mm	0.72 (0.58-0.82)	<0.001
Pre	Emin	Intralesional	0.42 (0.20-0.60)	<0.001
Pre	Emin	1 mm	0.39 (0.16-0.57)	0.001
Pre	Emin	2 mm	0.39 (0.16-0.57)	0.001
Pre	Emin	3 mm	0.41 (0.18-0.59)	<0.001
Post	Emin	Intralesional	0.51 (0.31-0.67)	<0.001
Post	Emin	1 mm	0.30 (0.07-0.50)	0.007
Post	Emin	2 mm	0.26 (0.02-0.47)	0.016
Post	Emin	3 mm	0.26 (0.02-0.47)	0.019
Pre	Esd	Intralesional	0.77 (0.64-0.85)	<0.001
Pre	Esd	1 mm	0.63 (0.28-0.80)	<0.001
Pre	Esd	2 mm	0.66 (0.35-0.82)	<0.001
Pre	Esd	3 mm	0.66 (0.36-0.81)	<0.001
Post	Esd	Intralesional	0.87 (0.80-0.92)	<0.001
Post	Esd	1 mm	0.82 (0.72-0.88)	<0.001
Post	Esd	2 mm	0.84 (0.74-0.90)	<0.001
Post	Esd	3 mm	0.83 (0.74-0.89)	<0.001

## 4 Discussion

In this study, SWE technology was employed to comprehensively evaluate the stiffness of lesions and surrounding tissues in 66 breast cancer patients before and after NAC (15). We evaluated SWE parameters including Emax, Emean, Emin, Esd, and their rates of change at various distances. After excluding ΔEmin due to uninterpretable percentage changes, the remaining parameters were analyzed. Statistical results revealed significant differences in the majority of these parameters between the effective and ineffective groups (16, 17). Specifically, after NAC treatment, the Emax, Emean, and Esd values within the lesion and at 1mm, 2mm, and 3mm surrounding distances were significantly lower than before treatment (11, 12, 18). Additionally, the corresponding areas under the curve (AUC) for these rates of change were all greater than 0.8, consistent with previous research findings (19). These discoveries suggest that measuring the stiffness of lesions and their adjacent regions can provide valuable guidance for NAC treatment plans in breast cancer patients.

It is important to acknowledge a key distinction regarding the study design. SWE measurements in this study were obtained at baseline and after completion of all NAC cycles, without intermediate assessments during treatment (e.g., after 2–3 cycles). Therefore, the findings validate SWE-derived stiffness changes as biomarkers that correlate with final pathological response, rather than as a genuinely early predictive tool that could guide mid-treatment regimen modification. This distinction is clinically meaningful: while the ability to predict eventual response at the end of NAC may inform postoperative management, it cannot address the need for early treatment adaptation in patients who are not responding to initial chemotherapy. Future prospective studies incorporating serial SWE assessments during early treatment cycles are warranted to determine whether SWE can fulfill the role of an early predictive biomarker.

Further analysis indicated that the AUC values of ΔEmax and its rates of change at different distances (ΔEmax-1mm, ΔEmax-2mm, ΔEmax-3mm) before and after NAC, were all greater than 0.9, showing more favorable diagnostic performance compared with ΔEmean and ΔEsd. This result is similar to the application of SWE in differentiating benign and malignant breast tumors, where Emax, representing the stiffness of the most challenging part of the lesson, demonstrates high diagnostic performance (20, 21). It is worth noting that all SWE parameter measurements are based on selecting the region of interest (ROI), which relies on the accurate delineation of tumor boundaries. Considering the biological characteristics of tumor tissues and their surrounding areas, our study focused on the lesion. It extended to 1mm, 2mm, and 3mm distances around it, capturing more comprehensive stiffness information of the lesion and its adjacent tissues.

Additionally, the study found that ΔEmax before and after NAC had the highest AUC value, with a sensitivity of 88.89% and a specificity of 85.71%. Although its specificity was slightly lower compared to ΔEmax-1mm, ΔEmax-2mm, and ΔEmax-3mm (all three had a specificity of 90.48%), DeLong’s test confirmed that the AUCs of intralesional and peri-lesional ΔEmax were not significantly different, indicating their comparable diagnostic performance. Thus, measurements at any of these locations may be clinically useful for evaluating NAC response. Therefore, a comprehensive analysis combining ΔEmax and its rates of change at different distances should be conducted to evaluate NAC response in breast cancer.

The stiff rim sign refers to the annular distribution of red or orange areas representing higher tissue hardness in the lesion boundary region on elastography images (22, 23). Studies have shown that breast malignant lesions can infiltrate their surrounding tissues, triggering connective tissue reactions, including fibrotic hyperplasia, lymphocyte infiltration, and increased microvascular density (24, 25). These changes collectively contribute to increased hardness of the tissues surrounding the tumor. Tumor tissues undergo complex changes during treatment, such as cell degeneration, necrosis, liquefaction, slowed proliferation, and tissue fibrosis. These changes make the lesion tissue softer, especially after NAC, where the hardness of the mass typically decreases by 30% to 90%, and correspondingly, its elasticity coefficient also changes.

Recent advances in magnetic resonance elastography (MRE) have also shown promise in this domain. A prospective in-human MRE trial demonstrated that tumor biomechanics assessed by MRE after the first cycle of chemotherapy could indicate treatment response, potentially enabling mid-course treatment modification (26). These findings highlight the value of elastography-based approaches and underscore the need for future investigations comparing the predictive performance of SWE and MRE in the early NAC setting. Furthermore, emerging applications of deep learning to SWE data have shown potential for pre-treatment prediction of treatment response in preclinical tumor models (27), suggesting a complementary direction for future research that integrates conventional SWE parameters with artificial intelligence-based approaches.

However, this study has several limitations. First, it was conducted at a single center with only 66 patients. The sample size did not permit reliable analyses by molecular subtype, although response to neoadjuvant chemotherapy may differ among subtypes. SWE was assessed only before treatment and after the final chemotherapy cycle; thus, these findings cannot establish its value for guiding treatment changes during chemotherapy. Second, the high apparent AUC for intralesional ΔEmax (0.941) requires cautious interpretation. Several correlated SWE parameters and measurement locations were examined, while the Youden-based cutoff and its sensitivity and specificity were determined and evaluated in the same cohort. This process may produce optimistic estimates that do not reproduce in independent patients. Neither internal resampling validation nor external validation was performed. The discrimination and cutoff reported here therefore require evaluation in an independent, adequately sized cohort before clinical use. Third, the 1-, 2-, and 3-mm peri-lesional measurements were obtained from overlapping regions of the same lesions and are therefore correlated. Benjamini-Hochberg false discovery rate correction was applied to the 12 between-group comparisons of percentage changes reported in **Tables 1** and **2**. Comparisons of absolute SWE values at individual time points were not adjusted for multiple testing and should be considered exploratory. The consistent findings across the three peri-lesional regions therefore do not constitute independent replication. Fourth, low baseline Emin values, frequently below 10 kPa, produced unstable and difficult-to-interpret percentage changes. We therefore omitted ΔEmin from the percentage-change comparisons and ROC analyses while retaining the absolute Emin measurements. This limits conclusions about treatment-related changes in Emin. Fifth, measurement reproducibility varied across SWE parameters. Inter-observer ICCs were relatively low for some measurements, particularly Emin (0.26-0.51) and pre-treatment peri-lesional Emean (0.44-0.48). Intra-observer repeatability and inter-observer agreement for the qualitative stiff rim sign were not assessed. Future studies should evaluate both quantitative measurement reliability and consistency in stiff rim sign interpretation.

In summary, this study demonstrates that changes in SWE parameters, particularly ΔEmax measured within the lesion and in the peri-lesional regions (1 mm, 2 mm, and 3 mm), and the disappearance of the stiff rim sign are associated with pathological response to NAC. These findings suggest that SWE-derived stiffness changes may serve as useful adjunctive biomarkers for post-treatment response evaluation. However, given the single-center design, limited sample size, and lack of interim measurements, these results should be considered exploratory. Multi-center prospective studies with larger cohorts and longitudinal SWE assessments during early treatment cycles are needed to validate the clinical utility of SWE for guiding NAC management in breast cancer patients.

## Data Availability

The raw data supporting the conclusions of this article will be made available by the authors, without undue reservation.
